# South African stakeholders’ knowledge of community-based rehabilitation

**DOI:** 10.4102/ajod.v8i0.484

**Published:** 2019-09-25

**Authors:** Sarah Rule, Anton Roberts, Pamela McLaren, Susan Philpott

**Affiliations:** 1 Disability Innovation Africa, Faculty of Health Sciences, University of Cape Town, Cape Town, South Africa; 2 CBR Education and Training for Empowerment (CREATE), Pietermaritzburg, South Africa; 3 Disability Action Research Team (DART), Howick, South Africa; 4 School of Education, College of Humanities, University of KwaZulu-Natal, Durban, South Africa

**Keywords:** community-based rehabilitation, disability inclusive development, survey, South Africa, role of persons with disabilities

## Abstract

**Background:**

Community-based rehabilitation (CBR) is a complex concept and strategy that has been implemented in diverse ways globally and in South Africa. Internationally, some stakeholders have described CBR as confusing, and this may influence implementation. A southern African study reports that there is insufficient evidence of the understanding of CBR in the region to influence training, policy and practice.

**Objectives:**

The aim of this study was to investigate South African stakeholders’ knowledge of CBR.

**Method:**

This article reports on an electronic survey that was part of a larger mixed methods study. Based on the sample of 86 respondents, descriptive statistics were used to analyse the quantitative data and thematic analysis for the qualitative data.

**Results:**

The majority of respondents had had exposure to CBR, but almost a quarter had no knowledge of the CBR guidelines and matrix. The results revealed varying knowledge concerning the key concepts of CBR, its beneficiaries and its funders. Respondents identified persons with disabilities as having a central role in the implementation of CBR. Problems with the visibility of CBR programmes were noted, as well as misunderstandings by many therapists.

**Conclusion:**

The implementation of CBR, and its goal of ensuring the rights of persons with disabilities, is negatively affected by the confusion attached to the understanding of what CBR is. The misunderstandings about, and lack of visibility of, CBR in South Africa may hinder its growing implementation in the country in line with new government policies.

## Introduction

Community-based rehabilitation (CBR) is a complex concept, approach and strategy. Since the initial conceptualisation of CBR, there have been many developments in the field, including a paradigm shift away from CBR being conceptualised as purely a rehabilitation and health-orientated strategy located in the community (Deepak et al. 2011; M’kumbuzi & Myezwa 2016; Rule, Poland & Gona 2008; Rule 2013; Wickenden et al. 2012). The evolutionary change of CBR to disability-inclusive development should have an impact on implementation, training and policy, but requires that CBR stakeholders should have up-to-date knowledge.

The current international conceptualisation of CBR in the CBR guidelines and matrix (World Health Organization [WHO] 2010) covers a broad range of components – health, education, livelihoods, social and empowerment. Community-based rehabilitation also includes features such as disability rights as encapsulated in the United Nations Convention on the Rights of Persons with Disabilities (UNCRPD) (United Nations 2006), poverty reduction and community development, all of which are encompassed in the term ‘disability-inclusive development’ in the CBR guidelines (WHO 2010).

Deepak et al. (2011) found that the CBR matrix fairly describes the practice of the vast majority of CBR workers who had multi-sectoral responsibilities from the projects on three continents that they studied. The shift from working on health issues alone to covering education, livelihoods, social promotion, inclusion and empowerment was described as challenging but necessary for Mongolian CBR workers (Como & Batdulam 2012). Several authors note that any CBR or disability-inclusive development project or programme is unlikely to work across all components and elements of the CBR matrix, although they may focus on more than one component (Cayetano & Elkins 2016; CREATE 2015). The CBR stakeholders involved in a Latin American study by Grech (2015:22) indicated a contribution of the CBR guidelines to be that of ‘broadening the areas of intervention beyond health and rehabilitation, driving attention towards the various areas and intersectionalities’.

Kuipers and Cornielje (2012) describe CBR as being dynamic in nature, and thus it cannot be defined in narrow terms. As Grech (2015:12) cautions, there is a ‘need to look at CBR as not only fluid, but also [as] a concept that needs to be continuously (re)defined.’ Accordingly, there is diversity in the way CBR is implemented globally.

Another aspect of the complexity of CBR is that several concepts can be interpreted in different ways. For example, the conceptualisation of *community* may imply a group sharing a particular environment, a coherent geographical space or a common interest or characteristic such as disability (Rule, Poland & Gona 2008). This has implications for who government and CBR personnel engage with in policy development and implementation of CBR.

The role of persons with disabilities in CBR has also evolved over time. Historically, they were often seen only as recipients of services. Since the ILO, UNESCO and WHO Joint Position Paper of 1994, CBR has been described as being implemented by persons with disabilities themselves, as well as their families and communities (ILO, UNESCO & WHO 1994). However, this theoretical stance is not always implemented in practice. For example, Ned and Lorenzo (2016) describe a situation in South Africa in which government officials had neither the conceptual understanding nor an attitude of willingness to mobilise youth with disabilities to participate in existing or new CBR programmes.

An issue of concern that adds to complexity in CBR is that of the costs, funding and sustainability of programmes. Community-based rehabilitation was initially promoted as a low-cost option to spread rehabilitation services to masses of underserved persons with disabilities, but the study by Grech (2015) indicates that this is not necessarily the case. Community-based rehabilitation may be state-funded as in Japan (Morita et al. 2013), or funded by international non-government organisations (INGOs), as in many countries in Africa. Changes in international donor funding priorities can lead to a reduction or re-direction of funds, and a consequent lack of sustainability or responsiveness of CBR programmes (Booyens, Van Pletzen & Lorenzo 2015).

In the light of these complexities, several studies around the globe have found that stakeholders describe CBR in its entirety or different components of CBR as confusing concepts (Deepak et al. 2011; Grech 2015; M’kumbuzi & Myezwa 2016; Morita et al. 2013). The study by M’kumbuzi and Myezwa (2016) was motivated by the lack of information on the conceptualisation of CBR in southern Africa. These authors found that stakeholders’ descriptions of CBR had barely moved from the 2004 era and that, on the whole, they did not incorporate the issue of rights as captured in the UNCRPD.

Of concern is the impact that this lack of clarity has on implementation of CBR. Morita et al. (2013) described the lack of understanding of CBR as a factor impeding its implementation in Japan. Similarly, authors writing about other contexts such as Mongolia (Como & Batdulam 2012), low- and middle-income countries in Asia and the Pacific (Cayetano & Elkins 2016) and South Africa (Lorenzo & Motau 2014) found poor knowledge of CBR to be a major barrier to the practice of CBR.

### Community-based rehabilitation in the South African context

Various CBR projects have been implemented around South Africa, each having different emphases and methods of implementation. Historically, the only government funding for CBR has been provided through various provincial Departments of Health, resulting in many CBR projects being situated within the health sector.

However, there is potential for this to change with the recent White Paper on the Rights of Persons with Disabilities (Department of Social Development 2015) addressing all sectors of government, as well as civil society. Of particular importance concerning CBR is pillar 4 of the White Paper, which promotes the availability of disability-specific services, including ‘Specialised and *community-based rehabilitation [own emphasis]*, habilitation and psychosocial support services’ (Department of Social Development 2015:84). The definition of CBR used in the glossary of the White Paper is drawn from the internationally recognised ILO, UNESCO and WHO (2004) joint position paper on CBR. However, the implementation matrix for the White Paper does not mention CBR at all; and the targets for community development seem to focus on accessibility only rather than full inclusion for persons with disabilities.

In 2016, the Department of Health (DoH) in South Africa released its *Framework and Strategy on Disability and Rehabilitation*. This document sees CBR as key to the DoH vision of providing ‘accessible, affordable, appropriate and quality disability and rehabilitation services’ to people with disabilities (DoH 2016:13). The document specifically includes the CBR matrix. The DoH acknowledges that coordinated action among intersectoral stakeholders is a salient feature of CBR if persons with disabilities are to attain independent functioning (DoH 2016). The centrality of the participation of people with disabilities in services is also emphasised ‘based on the principles of community-based rehabilitation and using a disability-inclusive developmental approach and evidence-based practice’ (DoH 2016:13). The document has yet to be implemented throughout the country.

Although these two recent guiding documents include mention of CBR, if the policies are to be implemented to benefit persons with disabilities, it is essential that stakeholders have knowledge of CBR as conceptualised in them. To date, there has been no systematic collection of information on the knowledge of CBR among stakeholders in South Africa.

The specific research question guiding this study was ‘What knowledge do CBR stakeholders in South Africa have of CBR?’ This study therefore seeks to contribute to an evidence base on the knowledge of CBR stakeholders. Such an evidence base can contribute to guiding the training of CBR stakeholders as well as to the implementation of policies and the practice of CBR and disability-inclusive development in South Africa.

## Research method and design

The study reported on in this article was part of a broader research project to examine CBR in South Africa using a mixed methods approach. The case studies that formed the qualitative aspect of the research are reported on elsewhere (CREATE 2015). The research was undertaken within a critical realist paradigm, which embraces the use of both qualitative and quantitative methodologies (Krauss 2005).

### Research design

To obtain a snapshot of the knowledge of different stakeholders in respect of CBR, this study used a survey that elicited largely quantitative data. Two of the 10 main survey questions sought to elicit qualitative data. In addition, two optional questions on demographic data were included for those who wished to participate further in the research or who could recommend CBR projects or programmes in South Africa to be contacted. The electronic survey was developed using SurveyMonkey software. For those who reported difficulties in accessing the survey via SurveyMonkey – particularly visually impaired respondents or those with irregular Internet access – the survey was converted to a Microsoft Word document and emailed to them. This enabled participants to download the survey and complete it offline. Visually impaired people were able to use screen reading software to read the Microsoft Word document. The survey was also conducted telephonically with some respondents.

### Development of the survey tool

The survey questions were based on the literature that highlighted important and contentious issues and definitions of CBR. The literature on the history of CBR globally and in South Africa informed the development of the response options. Questions 1, 2, 5, 7 and 8 of the survey questionnaire allowed for multiple responses (see Appendix 1).

### Sampling

The survey was distributed to 367 potential respondents from all nine provinces of South Africa. Initially, respondents were identified from existing databases for national electronic mailing lists in the disability and rehabilitation sectors, including the mailing list of Rural Rehab South Africa. Following the first round of distribution of the survey, snowball sampling (O’Leary 2017) was used to identify and contact additional respondents.

### Participant description

**FIGURE 1:**
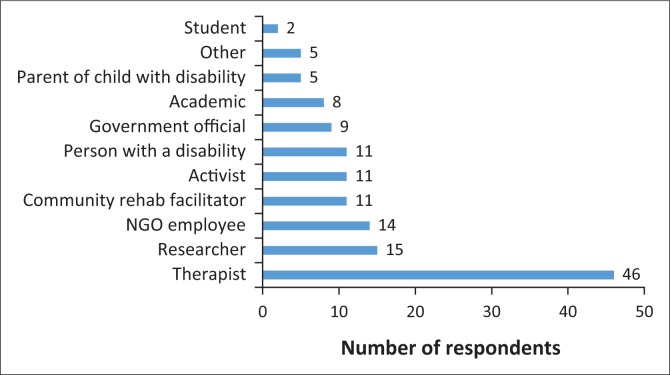
Number of respondents per category (multiple responses allowed).

In total, 86 people responded to the survey. Participants included the following: individuals from disabled people’s organisations; member organisations of the South African Disability Alliance; community rehabilitation facilitators; members of Rural Rehab South Africa; lecturers and staff from higher education institutions; NGO staff; government officials from the Departments of Health, Social Development and Education; and disability focal persons from other levels of government. Participants could self-identify in more than one category (Figure 1).

The question requesting demographic data was optional, which is a limitation of the survey tool as it prevents a comprehensive analysis of the geographic location of respondents. Only 53 of the 86 respondents identified the province they resided or worked in (Table 1) and no data was gathered on rural or urban location of respondents.

**TABLE 1:** Provincial and national representation of respondents (*N* = 53).

Province	*N*	%
KwaZulu-Natal	21	24.4
Gauteng	13	15.1
Eastern Cape	6	7.0
Western Cape	5	5.8
Mpumalanga	4	4.7
Northern Cape	1	1.2
North West	1	1.2
All provinces	2	2.3
No province indicated	33	38.4
Total	86	-

### Research procedure

The initial survey questionnaire was designed and piloted using SurveyMonkey, with eight targeted respondents taking part. Following analysis of the responses and comments in the pilot phase, the questionnaire was revised to improve the clarity of certain questions. The final questionnaire, together with an introductory letter for informed consent, was uploaded onto SurveyMonkey and then distributed as per the sampling described above. On analysing the summary data from initial responses collated by SurveyMonkey, the researchers realised that most of the respondents until that point were therapists, with disproportionally fewer community rehabilitation facilitators, disabled people’s organisations and persons with disabilities responding. Therefore, a second emailing of the survey specifically focused on the under-represented groups, along with telephone interviews conducted with seven community rehabilitation facilitators, some of whom were also persons with disabilities.

### Data analysis

In this study, the quantitative data were analysed using descriptive statistics. The SurveyMonkey software provided summaries of the quantitative data, which were exported into Microsoft Excel for analysis and calculation of frequencies and percentages. The qualitative data from Questions 9 and 10 of the survey (see Appendix 1) were analysed by a process of immersion in the data, ascribing meaning, coding themes and looking for interconnections (O’Leary 2017).

### Ethical considerations

Ethical considerations were catered for through three features of this study. Firstly, all participants received information about the nature and purpose of the study. Secondly, each participant provided informed consent, having been informed that they could refuse to answer particular questions or withdraw from the research by not returning the survey. Thirdly, no identifying information was linked to any individual responses to the survey, thus guaranteeing anonymity and confidentiality (O’Leary 2017).

## Results

The results of the survey are described in three subsections below, consolidating both quantitative and qualitative data.

### Exposure to and familiarity with community-based rehabilitation

**FIGURE 2:**
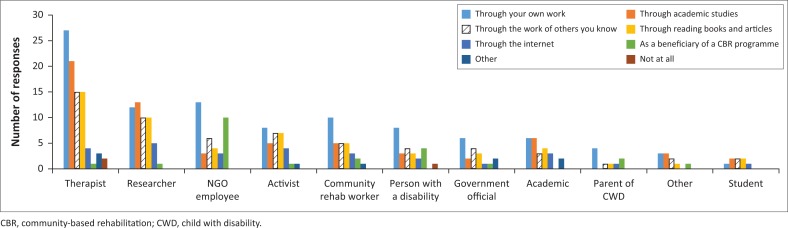
Cross-tabulation of respondent category with exposure to community-based rehabilitation (multiple responses allowed).

Participants were asked whether they had had any exposure to the practice or concept of CBR and, if so, from what sources. Only 3.5% of the respondents had never been exposed to CBR. The majority (68.6%) had been exposed through their own work, while almost half of the respondents (45.3%) had an awareness of CBR through their academic studies. Through cross-tabulation of questions 1 and 2 of the survey (Figure 2), it can be seen that for researchers the majority had exposure to CBR through academic studies, while for most other categories of respondents, their own work was the primary source of exposure to CBR.

**FIGURE 3:**
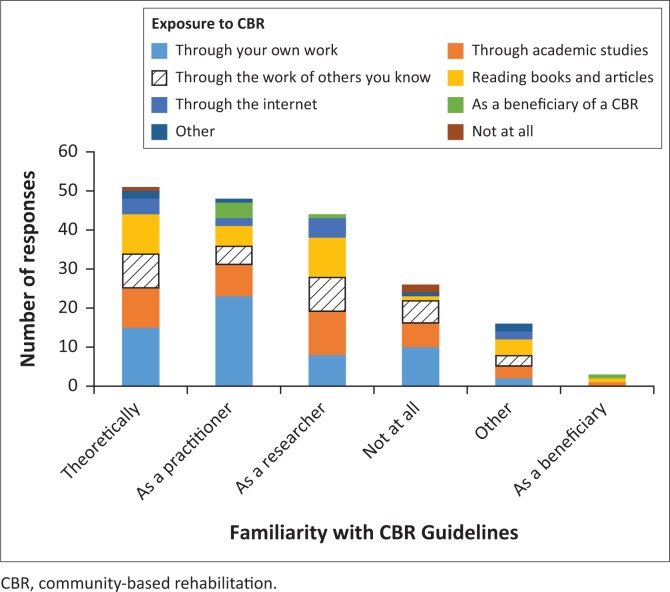
Cross-tabulation of respondents’ exposure to community-based rehabilitation (CBR) and familiarity with the CBR guidelines (multiple responses allowed).

This section was further expanded by asking participants about their familiarity with the World Health Organization’s (WHO’s) CBR guidelines and matrix (WHO 2010) (Figure 3). Only one response to this question was allowed. 23.3% of the respondents indicated that they had no knowledge of the CBR guidelines and matrix. When cross-tabulating questions 2 and 3 of the survey, it is unsurprising to find that the majority of those who were familiar with the CBR guidelines were also those who had been exposed to CBR through their own work. However, more than a third of those who were not at all familiar with the CBR guidelines were respondents who had been exposed to CBR in their own work.

### Knowledge of concepts underpinning community-based rehabilitation

To ascertain participants’ knowledge of CBR, the survey included five questions that looked at different concepts that are key characteristics of CBR. Question 6 of the survey concerned the definition of CBR. The options for the responses included different conceptualisations of CBR that have been in use internationally since the early 1980s, as well as some specific local South African enactments of CBR. A small majority of participants (50.6%) selected the response that CBR is ‘a programme that facilitates social inclusion and equal opportunities for persons with disabilities’. Only 17.6% of participants selected the response in line with the most recent international understanding of CBR as captured in the CBR guidelines (WHO 2010), being: ‘A strategy for disability-inclusive development’ (Table 2).

**TABLE 2:** Definitions of community-based rehabilitation selected by respondents (*N* = 85)

Definitions of CBR	*N*	%
A programme that facilitates social inclusion and equal opportunities for persons with disabilities	43	50.6
A strategy for disability-inclusive development	15	17.6
A rehabilitation strategy that offers intervention services to a particular community	11	12.9
A decentralised mobile outreach programme	10	11.8
A rehabilitation programme that offers peer counselling and basic services	6	7.1
**Total**	**85**	**-**

CBR, community-based rehabilitation.

Linked to the question concerning the definition of CBR, respondents were asked to select five of 12 listed activities that they felt were most important in CBR. All respondents answered the question (*N* = 86). The five most popular responses (Table 3) were involvement of persons with disabilities (84.9%); advocacy for disability rights (72.1%); rehabilitation (60.5%); inclusive development (58.1%) and community development work (43.0%). A key omission of the researchers was not to include ‘social inclusion’ as a response option.

**TABLE 3:** Important aspects of community-based rehabilitation (*N* = 86) (multiple responses allowed).

Activities that may be part of CBR	*N*	%
Involvement of persons with disabilities	73	84.9
Advocacy for disability rights	62	72.1
Rehabilitation	52	60.5
Inclusive development	50	58.1
Community development work	37	43.0
Referral to other resources	32	37.2
Education-related interventions	31	36.0
Outreach	28	32.6
Health-related interventions	26	30.2
Peer counselling	19	22.1
Poverty reduction	14	16.3
Other	2	2.3

CBR, community-based rehabilitation

Participants were asked about their understanding of the word ‘community’ in the context of CBR. The majority of respondents (55.3%) indicated that community refers to both a geographical community and those who have similar characteristics or interests.

The survey also explored the notion of beneficiaries of CBR programmes. Of the nine options, the most frequently selected responses were children and youth with disabilities (70.7%) and families of people with disabilities (68.3%), while the least frequently chosen beneficiaries were community leaders, community-based organisations and community members (Table 4).

**TABLE 4:** Beneficiaries of community-based rehabilitation programmes (*N* = 82) (multiple responses allowed).

Beneficiaries of a CBR programme	*N*	%
Children and youth with disabilities	58	70.7
Families of people with disabilities	56	68.3
Adults with disabilities	47	57.3
Parents or caregivers of children with disabilities	43	52.4
Marginalised and low-income people with disabilities	42	51.2
Disabled people’s organisations	31	37.8
Community-based organisations	21	25.6
Community members	21	25.6
Community leaders	8	9.8

CBR,Community-based rehabilitation

In this survey, participants were asked to select entities that they thought should fund CBR in South Africa (choosing as many as they liked from a list of 12 options). The results (*N* = 83) indicated that the most frequently selected entities were government departments. The NGO option for funding CBR is substantially lower than ‘government departments’ (Table 5).

**TABLE 5:** Funders for community-based rehabilitation programmes in South Africa (*N*= 83) (multiple responses allowed).

Potential CBR funder	*N*	%
Department of Health	80	96.4
Department of Social Development	74	89.2
Department of Education	66	79.5
Municipality	59	71.1
Department of Labour	51	61.4
NGO	35	42.2
Department of Cooperative Governance and Traditional Affairs	31	37.3
Disabled People’s Organisation	25	30.1
Academic institution	20	24.1
Organisation of parents of children with disabilities	18	21.7
Other	11	13.3
None of the above	1	1.2

CBR, community-based rehabilitation

### The role of persons with disabilities in community-based rehabilitation

Seventy-four of 86 participants responded to an open-ended question (question 9) concerning the role of persons with disabilities in CBR. Thematic analysis of the responses yielded three broad themes as described below.

### Requirements for the involvement of persons with disabilities in community-based rehabilitation

An NGO employee indicated that persons with disabilities must have ‘a role that ensures their voice is being heard and implemented in the CBR programme’. Expanding on the theme of prerequisites for the involvement of persons with disabilities in CBR, an employee of an NGO stated: ‘No one understands the needs of the person with disability as well as the person himself [sic]. It is vitally important that no decisions are made without their active involvement.’ Several respondents mentioned that persons with disabilities might need some training, education or skills development to play a role in CBR.

### Core principles of community-based rehabilitation

One academic, researcher and activist described the core role of people with disabilities as follows:

‘Engaged citizenship is a core feature of democracy…the UNCRPD, the World Report on Disability, the MDG’s etc. and other policy guidelines reiterate the deconstruction of “professional” hegemony… how can any “project” (which in itself is NOT CBR) even be conceived without PWD taking the forefront?’ (Participant 43, academic, activist)

### Actual roles of persons with disabilities in community-based rehabilitation programmes

Many participants identified specific roles of persons with disabilities in CBR programmes. These roles included involvement throughout the life cycle of the programme, from planning and designing to implementation, monitoring and evaluation. Also taking a role in designing CBR programmes and monitoring them, persons with disabilities were clearly seen as beneficiaries in a more or less active role:

‘The agenda, needs and concerns of disabled people are as diverse as any other “group” and this is a challenge particularly if the beneficiaries choose to lead projects themselves. Consulting with users/beneficiaries is an important part of any service, project etc. though.’ (Participant 80, researcher, therapist)

Several respondents mentioned advocacy and awareness-raising as an important role for persons with disabilities: ‘As beneficiaries they need to advocate for themselves to different Govt. [government] departments to access their rights’ (Occupational Therapy Technician). Another common response was that persons with disabilities should engage in peer support or counselling, and form their own support groups within a CBR programme. An important role of persons with disabilities was described as follows: ‘They should be watchdogs – make sure that funding goes to the people that need the rehabilitation and not to the organisations that do it’ (person with a disability who is also a parent of a disabled child).

Some respondents indicated that the various active roles that persons with disabilities play in CBR programmes would lead to sustainability of those programmes.

#### General comments on community-based rehabilitation in South Africa

The final open-ended question in the survey elicited general comments on CBR in South Africa illustrating participants’ knowledge of CBR. Fifty-two out of 86 participants (60.5%) responded to the question with a wide range of comments and issues.

### Community-based rehabilitation in the context of South Africa

A researcher and therapist was particularly concerned about the efficacy of CBR in the South African context of poverty and unemployment:

‘I have concerns that the attitudinal shift re: disability that CBR tries to address is a bigger societal issue than individual CBR projects can address. For example, disabled children will never be truly included in mainstream education whilst mainstream education is being so poorly provided generally, and perhaps the same kind of principle applies when encouraging disabled people to voice their needs and uplift themselves in areas where there is a much bigger picture of poverty, lack of schooling, lack of jobs etc.’ (Participant 80, researcher, therapist)

The national context of historical contestations on the implementation of CBR was also of concern to a government official:

‘The debate on CBR in South Africa is derailed by a narrow definition of CBR limiting it to the cadre that should provide it and not the conceptual underpinnings of the strategy. Far too often the responsibility has been placed under Health whereas all literature is clear on the cross-cutting nature of the strategy.’ (Participant 84, government official, national)

### The status of community-based rehabilitation in South Africa

Several respondents were concerned that CBR projects or programmes were either not visible or did not exist in various areas of South Africa. As a community rehabilitation facilitator put it:

‘I have struggled to come across practical examples of how CBR has been implemented in South Africa. Completely underestimated and not publicized enough. People who are not in the health sector don’t know about CBR.’ (Participant 20, therapist, KwaZulu-Natal)

This may explain why the majority of respondents to the survey were therapists, working in the health sector. Lack of visibility may contribute to confusion about CBR that seven of the respondents identified. For example:

‘There is mass confusion amongst rehab professionals about CBR, and the term is often loosely used to refer to any rehab service rendered by a professional outside the 4 walls of a hospital.’ (Participant 24, therapist, activist)

#### Barriers to the implementation of community-based rehabilitation

The barriers are closely linked to the status of CBR in South Africa and the context of the programmes. For instance, the misunderstanding of CBR, as illustrated above, undermines its implementation, as does the lack of visibility. Resource constraints and poor management also impact negatively on the implementation of CBR:

‘All our DPOs *[disabled people’s organisations]* and DPO projects died a slow death while the members were waiting for the money municipality promised them for the projects… Those who started *[the projects]* died because of internal fights regarding the management of the donated funds. (Therapist)’ (Participant 52, therapist, KwaZulu-Natal)

#### The value of community-based rehabilitation

Nine respondents wrote of the difference that CBR could make in the lives of persons with disabilities. One respondent, a researcher, community rehabilitation facilitator and activist related the value of CBR specifically to the empowerment component of the CBR matrix:

‘I believe that the empowerment component of CBR can be a fantastic, cost-effective intervention. I also think that a focus on human rights literacy is essential for holding State services accountable for providing accessible and adequate services.’ (Participant 51, community rehabilitation facilitator, activist)

#### Opportunities and recommendations for community-based rehabilitation in South Africa

A number of responses centred on using opportunities for CBR that currently exist in South Africa, with the development of national health insurance and the *Framework and Strategy for Disability and Rehabilitat ion* (DoH 2016). Recommendations were made particularly for the development of human resources in the field of CBR, including government officials, CBR workers and CBR managers. A CBR worker made a specific recommendation for higher education institutions:

‘CBR is a good strategy in South Africa, and it should be introduced in detail in higher education, meaning departments that train students to work with persons with disabilities must introduce this concept at an earlier age – not when students are coming for their practical or community block that is only four or six weeks.’ (Participant 87, community rehabilitation facilitator, Gauteng)

## Discussion

The main quantitative findings showed that three-quarters of respondents had some knowledge of CBR, with half attributing this to their academic studies. However, almost a quarter indicated that they were unfamiliar with the CBR guidelines and matrix (WHO 2010). Half of the respondents conceptualised CBR as the facilitation of social inclusion and equal opportunities for persons with disabilities, but only 17.6% viewed it as ‘a strategy for disability-inclusive development’. Both advocacy for disability rights and inclusive development featured in the most commonly selected aspects that make up CBR. Children and youth with disabilities, as well as families of people with disabilities, were cited most commonly by respondents as beneficiaries, while a quarter saw beneficiaries as being community members and community-based organisations. Almost all respondents indicated that funding of CBR is the responsibility of government departments, while just over 40% indicated that NGOs should also fund it. The qualitative data revealed a focus on the essential requirement for CBR being the involvement of persons with disabilities, with the core principle that persons with disabilities have a key role to play in decision-making. This involvement needs to occur throughout the life cycle of the programme, including design and monitoring phases. In addition to advocacy and awareness-raising, peer support was seen as a key role to be played by persons with disabilities. Respondents identified a range of factors that undermine CBR within the wider context of South Africa, including its perception as a health-focused intervention, its lack of visibility and the absence of nationwide implementation. Thus, despite the potential value of disability-inclusive development for the empowerment of persons with disabilities, and opportunities emerging from current policy developments, systemic challenges remain, particularly in respect of human resources.

The following themes are seen to be relevant to the study findings on the knowledge of CBR in South Africa.

### Profile of participants

Analysis of results needs to take into account the profile of respondents of this study, particularly the fact that over half (53.5%) identified themselves as being therapists. The authors acknowledge that the skewed sample could be attributed to a limitation of this study, namely the method of recruiting participants, being an electronic survey of various stakeholders, including those reached through the database of Rural Rehab South Africa. As a result, very few persons with disabilities and parents of children with disabilities were respondents, a critical limitation given the central role that they have to play in disability-inclusive development.

### Knowledge of community-based rehabilitation and source thereof

From the responses received through the survey, it is evident that respondents seem to have moved away from the conceptualisation of CBR as predominantly dealing with health and rehabilitation issues. However, participants’ knowledge of CBR does not reflect international developments in the field, particularly in respect of the definition of CBR as a strategy for disability-inclusive development, which captures the conceptualisation of CBR in the guidelines (WHO 2010). This study confirms that academic and training institutions are a key source of knowledge about disability-inclusive development, and thus the equipping of personnel with the necessary skills, knowledge and attitudes is a challenge that they need to address. Furthermore, because awareness is also gained through exposure in their own work, the practice of disability-inclusive development needs to be infused into all relevant sectors. This may be supported using tools from the disability and development fields that are based on the WHO CBR guidelines, for example in programme evaluation and funding frameworks.

### Beneficiaries

In this study, the majority of respondents saw the primary beneficiaries of CBR as being persons with disabilities and their families. The least frequently chosen beneficiaries of CBR programmes were community leaders, community members and community-based organisations. This may be related to the phrasing of the question in the survey. It may also indicate an aspect of the conceptualisation of CBR that needs to be discussed and developed further among stakeholders. In the study by Booyens, Van Pletzen and Lorenzo (2015), community disability workers specifically highlighted the importance of working with traditional community leaders to influence their attitudes towards persons with disabilities and improve their social inclusion. Disability-inclusive development cannot be achieved without the involvement of the broader community and therefore it is essential that if the *Framework and Strategy for Disability and Rehabilitation* (DoH 2016) is to be fully implemented in South Africa, stakeholders must understand the role and value of community leaders and other community members.

### Role of government and civil society

Various iterations of CBR by the ILO et al. since 2004 have included the need for collaboration between different sectors, such as health and education. In addition, both government and NGO actors reportedly have roles to play in disability-inclusive development. In some countries, there are state-funded CBR programmes from different sectors, while in other countries CBR is mainly funded by international (and sometimes local) NGOs. In discussing the sustainability of CBR, the CBR guidelines (WHO 2010) compare the relative benefits and drawbacks of government-supported or government-led CBR programmes with those led or supported by civil society. While government-supported programmes may make community participation and ownership more difficult, they can provide a more stable source of funding that is also important for sustainability. In response to the question of who should fund CBR in South Africa, the vast majority of responses indicated that a variety of different government departments should do so. This may reflect the reality of the difficult funding climate for civil society organisations in South Africa.

With the development of national health insurance in South Africa, there is an opportunity for some provision of disability-inclusive development to be covered by the DoH. However, there is the danger that programmes funded through national health insurance will focus on the health component, to the exclusion of work in the education, livelihood and, to some extent, social and empowerment components of CBR, as these do not fall within the line functions of the DoH. Iemmi et al. (2016) describe CBR programmes as those that have interventions in one or more of the components of the CBR matrix and respond flexibly to the needs of the users of the service. Currently, there is no funding model for disability-inclusive development in South Africa across different government departments. If CBR is only funded through the DoH, disability-inclusive development programmes could well struggle to be flexible and diverse, and to have a holistic approach to persons with disabilities.

### Role of persons with disabilities

The maxim ‘nothing about us without us’ is central to the UNCRPD and the White Paper on the Rights of Persons with Disabilities, as well as being a premise on which the CBR guidelines are based. While the majority (84.9%) of respondents expressed knowledge of the importance of the role of persons with disabilities in disability-inclusive development, it was not possible to establish the extent to which such involvement actually materialises. Indeed, the methodology of this study itself illustrates some of the challenges of ensuring such involvement. It is therefore essential to move beyond the ‘knowledge’ that persons with disabilities and their families need to be key decision-makers and shapers of disability-inclusive development, to fostering the necessary values, skills and structures to ensure that this actually happens in a meaningful way. This implies that training programmes and tertiary institutions equipping participants in disability-inclusive development need to include in their curricula not only inputs from persons with disabilities and parents of children with disabilities, but also the skills to facilitate such involvement.

### Factors impeding disability-inclusive development

In 2006, Rule, Lorenzo and Wolmarans observed a lack of visibility of existing CBR programmes and a lack of published data on what works and what does not work in the South African context. They identified the urgent need to raise the profile of CBR in South Africa. Unfortunately, several comments in the survey conducted 11 years later indicated that this has not yet been achieved.

While many of the respondents in this study had knowledge of CBR, there are many additional issues that need to be addressed if disability-inclusive development is to be implemented nationally as part of the White Paper on the Rights of Persons with Disabilities (Department of Social Development 2015) and the *Framework and Strategy on Disability and Rehabilitation* (DoH 2016). One such issue is the question as to whether disability-inclusive development is only for the disability community or whether it applies more broadly. It will be important to promote an understanding of disability-inclusive development as a broad strategy addressing empowerment, livelihoods, education and social aspects, as well as health and rehabilitation. This also impacts government, which currently has no mechanism or provision for intersectoral budgeting for disability-inclusive development in South Africa.

## Limitations of the study

There were two major limitations of this study: firstly, this was a rather simple survey that was not representative. The majority of the participants in the research were therapists and only a small number were persons with disabilities and parents of children with disabilities. Use of the database of Rural Rehab South Africa to recruit respondents meant that mainly therapists were targeted by the survey. Furthermore, the mode of response to the survey (requiring Internet or email access) is likely to have limited the number of respondents from categories other than therapists, academics and researchers. Secondly, the questionnaire was not tested for reliability and validity.

A means of addressing these limitations could be to set up a comprehensive database of disability-inclusive development stakeholders in South Africa, from which sampling could take place in further research. In addition, future studies should use telephonic interviews to help overcome the limitation of the representativeness of the sample, as many of the under-represented groups may not have had access to email or have felt comfortable to express themselves in writing in response to the questions raised.

It is important to identify what was measured in this study. The main ambition was to map certain aspects of stakeholders’ knowledge of CBR in South Africa. The focus was on knowledge of information and facts while recognising that it was not possible to assess the understanding or application of this knowledge through an electronic survey. It is thus recommended that further in-depth research should be conducted, using methods such as case studies to document how current knowledge of disability-inclusive development manifests in practice. It is recognised, however, that knowledge and understanding of disability-inclusive development are not the only factors that shape service provision. Other factors, including training and resource allocations, can enhance or impede its implementation.

## Conclusion and recommendations

The main message from this study is that the implementation of CBR in South Africa, and through CBR ensuring the rights of persons with disabilities, is negatively affected by the confusion attached to the understanding of what CBR is in South Africa.

In their literature review, Cayetano and Elkins (2016) claimed that one of the major barriers to CBR in the Asia-Pacific region has been the lack of understanding of CBR by professionals and CBR workers, and specifically a misunderstanding of the purpose of CBR. While many participants in this study demonstrated at least some understanding of CBR in the form conceptualised in the CBR guidelines, there is still a lack of consistency in their responses regarding the nature of CBR.

To overcome barriers and ensure the implementation of disability-inclusive development in South Africa to promote human rights and the implementation of the UNCRPD and the White Paper on the Rights of Persons with Disabilities (Department of Social Development 2015), the authors recommend building greater awareness of CBR as conceptualised in the CBR guidelines through:

- Publicising and promoting working models of CBR in South Africa that adhere to the CBR guidelines.
- Providing training on CBR to stakeholders already in the field, including disabled people’s organisations, organisations of parents of children with disabilities, service providers and government officials.
- Ensuring that all academic and practical training of therapists, social workers and other professionals includes up-to-date conceptualisations of CBR.

## Appendix 1: Community-based rehabilitation: Understanding it in South Africa

Community-based rehabilitation (CBR) has historically been an aspect of rehabilitation in the health sector in South Africa. Internationally, CBR has undergone a number of changes, but we have no data on how CBR is currently understood in South Africa. By responding to this survey, you will be assisting us to determine how people in South Africa understand CBR now.

In this survey you will find questions that are optional as well as those to which responses are required (marked with an asterisk). By completing the survey you are giving consent for your responses to be included in the results. However, CREATE undertakes to ensure that your identifying information will not be linked to your responses nor will it be revealed to anyone except the researchers.

*1. Which of these terms describes you best? (Select all applicable)
  □ Person with a disability
  □ Parent of a child with a disability
  □ Student
  □ Academic
  □ Researcher
  □ Therapist
  □ Community rehabilitation facilitator or worker
  □ Government official
  □ Activist
  □ Employee of a non-government organisation
  □ Other (please specify)
*2. Have you ever been exposed to the concept and/or practice of community-based rehabilitation in South Africa?
  □ Not at all
  □ Through your own work
  □ Through the work of others you know
  □ As a beneficiary of a community-based rehabilitation programme
  □ Through academic studies
  □ Through the Internet
  □ Through reading books and articles
  □ Other (please specify)
*3. Are you familiar with the World Health Organization’s CBR guidelines and CBR matrix?
  □ Not at all
  □ Theoretically
  □ As a practitioner
  □ As a beneficiary of a community-based rehabilitation programme
  □ As a researcher
  □ Other (please specify)
4. Which definition of community is most appropriate for community-based rehabilitation in South Africa?
  □ A group of people with common interests or characteristics, for example, disability
  □ A group of people who live in a particular geographic area
  □ Both of the above
  □ Other (please specify)
5. In your understanding, what are or would be the five most important aspects of community-based rehabilitation?
  □ Inclusive development
  □ Involvement of people with disabilities
  □ Peer counselling
  □ Health-related interventions
  □ Advocacy for disability rights
  □ Education-related interventions
  □ Rehabilitation
  □ Outreach
  □ Referral to other resources
  □ Community development work
  □ Poverty reduction
  □ Other (please specify)
*6. Please select the statement that most closely represents your view of community-based rehabilitation.
  □ A decentralised mobile outreach rehabilitation programme
  □ A rehabilitation strategy that offers intervention services to a particular community
  □ A strategy for disability inclusive development
  □ A rehabilitation programme that offers peer counselling and basic skills
  □ A programme that facilitates social inclusion and equal opportunities for persons with disabilities
  □ Don’t know
  □ Other (please specify)
7. Who should be the beneficiaries of a community-based rehabilitation project or programme? Select the four most important beneficiaries.
  □ Community leaders
  □ Marginalised and low income people with disabilities
  □ Community-based organisations
  □ Children and youth with disabilities
  □ Adults with disabilities
  □ Families of people with disabilities
  □ Parents or caregivers of children with disabilities
  □ Community members
  □ Disabled people’s organisations
  □ Other (please specify)
8. If there are to be community-based rehabilitation projects or programmes in South Africa, who should fund them? (Select as many responses as applicable)
  □ Department of Health
  □ Department of Education
  □ Department of Labour
  □ Department of Cooperative Governance & Traditional Affairs
  □ Department of Social Development
  □ Municipality
  □ Disabled People’s Organisation
  □ Organisation of parents of children with disabilities
  □ Non-government organisation
  □ Academic institution
  □ None of the above
  □ Other (please specify)
9. What role should persons with disabilities play in a community-based rehabilitation project?
10. Please record any comments you have on community-based rehabilitation in South Africa.
11. Please supply your contact details if you wish to participate further in the research on community-based rehabilitation in South Africa.
  Name:
  Province:
  Email address:
  Phone number:
  We would like to get a picture of community-based rehabilitation throughout South Africa. We would appreciate it if you could direct us to any CBR projects or programmes you know of.
12. Contact details for community-based rehabilitation projects or programmes in South Africa Name of CBR project or programme:
  Province:
  Email address:
  Phone number:

**Thank you for assisting us by completing this survey. Please contact CREATE with any queries at create3@telkomsa.net.**
